# A Gradient-Based Approach for Breast DCE-MRI Analysis

**DOI:** 10.1155/2018/9032408

**Published:** 2018-05-16

**Authors:** L. Losurdo, T. M. A. Basile, A. Fanizzi, R. Bellotti, U. Bottigli, R. Carbonara, R. Dentamaro, D. Diacono, V. Didonna, A. Lombardi, F. Giotta, C. Guaragnella, A. Mangia, R. Massafra, P. Tamborra, S. Tangaro, D. La Forgia

**Affiliations:** ^1^I.R.C.C.S. “Giovanni Paolo II” National Cancer Centre, Bari, Italy; ^2^Department of Physics, University of Bari “Aldo Moro”, Bari, Italy; ^3^National Institute for Nuclear Physics (INFN), Bari Division, Bari, Italy; ^4^Department of Physical Sciences, Earth and Environment, University of Siena, Siena, Italy; ^5^University of Bari “Aldo Moro”, Bari, Italy; ^6^Electrical and Information Engineering Department, Polytechnic University of Bari, Bari, Italy

## Abstract

Breast cancer is the main cause of female malignancy worldwide. Effective early detection by imaging studies remains critical to decrease mortality rates, particularly in women at high risk for developing breast cancer. Breast Magnetic Resonance Imaging (MRI) is a common diagnostic tool in the management of breast diseases, especially for high-risk women. However, during this examination, both normal and abnormal breast tissues enhance after contrast material administration. Specifically, the normal breast tissue enhancement is known as background parenchymal enhancement: it may represent breast activity and depends on several factors, varying in degree and distribution in different patients as well as in the same patient over time. While a light degree of normal breast tissue enhancement generally causes no interpretative difficulties, a higher degree may cause difficulty to detect and classify breast lesions at Magnetic Resonance Imaging even for experienced radiologists. In this work, we intend to investigate the exploitation of some statistical measurements to automatically characterize the enhancement trend of the whole breast area in both normal and abnormal tissues independently from the presence of a background parenchymal enhancement thus to provide a diagnostic support tool for radiologists in the MRI analysis.

## 1. Introduction

Breast Magnetic Resonance Imaging (MRI) has been shown to have higher sensitivity than mammography and ultrasonography in women at increased risk for breast cancer and is increasingly being used for screening purposes [1–3]. While baseline breast MRI before the administration of the contrast agent can be used to measure the extent of fibroglandular tissue [4, 5], the use of enhancement on MRI after intravenous administration of a gadolinium chelate represents a sensitive method to describe in vivo physiologic and biologic activities in breast tissue which are related to breast cancer risk [6, 7]. Indeed, the time-signal intensity curve (or kinetic curve) in breast Dynamic Contrast Enhancement MRI (DCE-MRI) reflects the hemodynamic features of a specific lesion: the initial rise usually reflects the extent of tumor angiogenesis, whereas the delayed phase reflects the formation of stromal tumor cells. Generally, a* persistent* curve is suggestive of benign changes,* washout* is related to malignancy, and* plateau* can represent either benign change or malignancy [8, 9].

However, in some cases it can happen that the normal tissue can take contrast as well as the abnormal one. This phenomenon of contrast enhancement of the normal breast tissue in DCE-MRI is known as background parenchymal enhancement (BPE); it may represent breast activity and depends on several factors (including tissue vascularity and permeability, endogenous and exogenous hormones, and endocrine therapy effects) [10–12]. It is assessed by four qualitative Breast Imaging-Reporting and Data System (BI-RADS) categories: minimal, mild, moderate, and marked [13]. However, although clinically useful, BI-RADS-based BPE assessment is subjective with high intra- and interreader variability.

Despite its high sensitivity, breast DCE-MRI requires standardized acquisition protocols and longer time for image processing and interpretation and has variable specificity. Indeed, the evaluation of a large quantity of 4D-DCE images for each patient is a time-consuming process, and their interpretation requires experienced radiologists [14]. In literature, textural analysis techniques have been applied to dynamic breast MRI to quantify BPE [15], discriminate malignant from benign tissue [16–18], or identify particular topology of lesions [19]. Therefore, the focus of the main works at the state of the art regards diagnostic studies of diseases. Nevertheless, before the characterization phase, the identification of lesions can be hard and radiologists may have difficulty distinguishing such lesions especially with a diffuse and moderate or marked BPE [10, 20–22]. For these reasons, fully automated computerized approaches to MRI analysis able to extract informative knowledge characterizing the whole breast area enhancement trend and give relevance to specific breast regions with respect to others, even in case of diffuse BPE, are of great clinical importance [6, 23, 24].

Usually, the shape of the time-signal intensity curve represents an important measure in discriminating benign and malignant enhancing lesions, and automatic approaches reported in literature perform automated image processing and quantitative analysis of contrast uptake on lesions to improve observer reproducibility in DCE-MRI [1, 25, 26]. Specifically, in contrast to conventional manual region of interest segmentation approaches, they generate detailed kinetic data for all pixels in the lesion and can provide quantitative whole-lesion evaluation. Moreover, most of them evaluate the approach on mass-like lesions without considering non-mass-like enhancing lesions (according to the BI-RADS breast MRI lexicon, a mass is a 3D space-occupying lesion. Comparatively, the enhancement of an area that is not a mass refers to a non-mass-like enhancing lesion. The detection of non-mass-like enhancing lesions is an important question, because a large number of breast lesions have non-mass-like enhancement).

The aim of this study is to investigate the utility of some statistical measurements of DCE to automatically characterize normal and abnormal tissues, independently from the BPE degree and the lesion morphological shape. The proposed approach can provide a diagnostic support tool for radiologists in the MRI analysis to highlight suspicious breast regions that require further evaluation. Specifically, we identified the gradient and the entropy, evaluated on the temporal DCE-MRI scans, as two measures to synthetically represent the enhancement evolution over time and the mean and standard deviation as further criteria to distinguish a lesion with respect to the other breast structures regardless of the type of breast/lesion morphology.

## 2. Materials and Methods

### 2.1. Materials

For this preliminary study, we have collected 46 breast MRI scans in DICOM format from women who underwent breast MRI examination in I.R.C.C.S. “Giovanni Paolo” II (informed consent is waived) in Bari from 2014 to 2017. Among them, 23 individuals were without any lesions on MRI or mammography or ultrasound examination. The analyzed MRI findings belong to patients aged between 23 and 75 years, with an overall average age of 48.02 ± 10.28 years. Specifically, 19 patients (41.3%) have an age <45, the same number and percentage for the patients aged between 45 and 55, and 8 (17.4%) patients have an age > 55. MRI diagnostic questions were screening in high-risk patients and diagnosis of multifocal/multicenter carcinomas or suspicious abnormalities in previous mammogram and/or ultrasound for patients with dense breast.

Breast MRI scanning process was performed in the prone position with a dedicated seven-channel breast coil on a 1.5 Tesla PHILIPS scanner (Achieva®, Philips Medical Systems, Best, Netherlands). In premenopausal patients, MRI was performed in the second week of menstrual cycle. A short T1 inversion recovery (STIR) turbo-spin-echo (TSE) sequence, a T2-weighted TSE sequence, and a 3D-DCE T1-weighted sequence were acquired. The complete MRI acquisition parameters are reported in Table 1. Specifically for the 3D-DCE T1-weighted sequence, six dynamic acquisitions were performed, resulting in 1.5 mm^3^ isotropic voxels; time of dynamic data acquisition was 63 s and the total sequence duration was 9 min. 150 axial slices for the six sequences were utilized to cover the entire breast, one scan before the administration of contrast agent and five scans after an intravenous injection of gadobenate dimeglumine (MultiHance®, Bracco, Milan, Italy), at a dose of 0.1 mmol/kg of body weight and flow rate of 1.5 mL/s followed by 20 mL of saline solution. After the dynamic series, a process of subtraction of the MR images between the postcontrast dynamic sequences and the precontrast one is automatically performed: in this way, other images may be visualized, called* subtractions* or* subtracted images*, where the lesions with enhancement are emphasized. Then, a diagnostic workstation with dedicated software for MR imaging examination (View-Forum R5.1 V1L1 2006) analyzed all MRI images. An expert radiologist, with more than 15 years of experience in breast DCE-MRI examination and diagnosis, retrospectively annotated the lesions in a dedicated breast MRI database.

The cases affected by pathologies were analyzed by using a multimodal classification system defined for contrast-enhanced MRI lesions considering morphology and dynamics of contrast enhancement [27]. The morphologic evaluation criteria concerned the shape and the margin of the lesion, while the pattern of contrast medium (CM) enhancement within the lesion was evaluated as homogeneous/heterogeneous/rim. Regarding the dynamic aspect, the profile of the time-signal intensity curve was analyzed. The initial enhancement is the signal curve from the precontrast measurement to the maximum value in the first 3 min after the administration of CM, whereas from the maximum peak in the first 3 min to the end of the examination is defined as the postinitial enhancement [28]. Three types of postinitial curves were defined: continuous (increase > 10%), plateau (deviation of the signal curve between +10% and −10%), and washout (decrease > 10%). The criteria used for the estimation of the appropriate score are presented in Table 2. Depending on the correlation between the described evaluation criteria and the probability of malignancy, from 0 to 2 points were given for each criterion. A total of 0–8 points were assigned to each lesion. The point score provided a classification of the lesions according to the five categories as reported in Table 3. Specifically, for our sample of patients affected by pathology, 4 cases (17.4%) were classified as benign (class I) and 3 (13%) as probably benign (1 belonging to class II and 2 to class III); 8 patients (34.8%) had lesions with suspicious abnormality (class IV) and 8 patients (34.8%) had highly suggestive lesions of malignancy (class V). Note that the results reported in this preliminary study are actually binary (normal versus abnormal); however, in view of a future diagnostic work, our radiologists have also indicated the group to which each lesion belonged.

**Figure 1:**
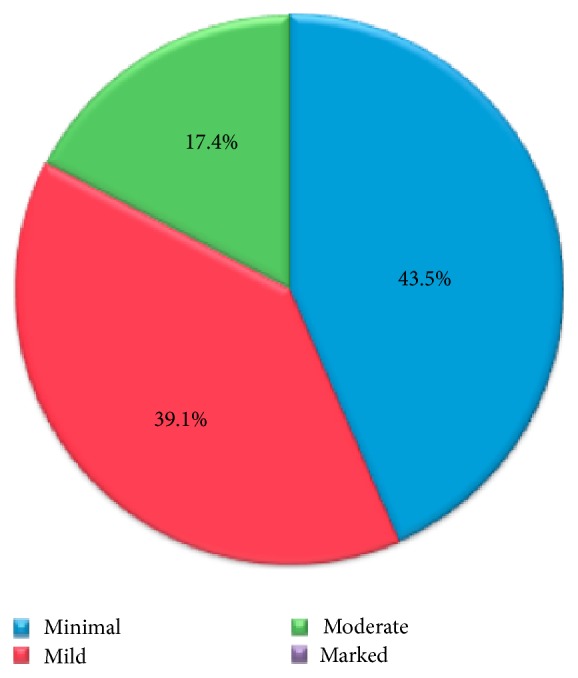
BPE categories distribution on the data.

Moreover, BPE was evaluated on subtracted MR images and classified as minimal (<25% of glandular tissue enhancement), mild (25–50% enhancement), moderate (50–75% enhancement), and marked (>75%), according to ACR BI-RADS [13, 29]. The distribution of the sample according to the BPE classification on the whole set of data is shown in Figure 1 (marked value is 0% in this set of data).

### 2.2. Methods

In this study, the gradient of the image and the entropy were evaluated as measurements to extract helpful information from the dynamic MRI acquisitions able to catch the enhancement dynamics.

As to the gradient, it can be used to extract information from images, since it is able to grasp the directional change in the intensity. Indeed, gradient images are usually created from the original ones for this purpose as each pixel of a gradient image measures the change in intensity of that same point in the original image in a given direction. The most common way to approximate the image gradient is to convolve the image with a kernel, that is, adding each element of the image to its local neighbours weighted by the kernel. One of the simplest and most used approaches for this task is the Sobel operator [30]. In particular, as we are interested in analyzing the image changes during the MRI dynamic acquisitions, Sobel was applied to derive the directional gradient in the third dimension that represents the time-step of the scans. This process is configured as a 3D convolution on a 3 × 3 × 3 kernel computation. Specifically, for the directional gradient in the time dimension *z*, the kernels exploited by Sobel operator are

(1) Kz:,:,−1=+1+2+1+2+4+2+1+2+1,Kz:,:,0=000000000,Kz:,:,1=−1−2−1−2−4−2−1−2−1.

Calculating such a convolution could result to be computationally expensive. However, noting that Sobel operator consists of two separable operations, which are smoothing perpendicular to the derivative direction with a triangle filter (*h*(−1) = 1, *h*(0) = 2, and *h*(1) = 1) and performing the central difference in the derivative direction (*h*′(−1) = 1, *h*′(0) = 0, and *h*′(1) = −1), it is sufficient to apply the 1D filter (*h* and/or *h*′) in each direction consecutively. In particular, the 1D *h*′ filter should be applied in the direction one is interested in. Hence, the directional gradient in time dimension *z* results:

(2) $$\begin{array}{c} h_{z}^{\prime }\left(\;x,y,z\;\right)=h\left(\;x\;\right)h\left(\;y\;\right)h^{\prime }\left(\;z\;\right). \end{array}$$

Regarding the entropy, it is a statistical measure of randomness which can be used to characterize the texture of the input image. The entropy of an image *I* is defined as

(3) $$\begin{array}{c} E\left(\;I\;\right)=-\overset{N}{\underset{k=0}{\sum }}p_{k}\;\log_{2}\;p_{k}, \end{array}$$

where *p*_*k*_ = Pr⁡(*x* = *k*), the probability of an image pixel *x* to assume the value *k*, and *N* = 255, that is, the gray levels of the image slice.

**Figure 2:**
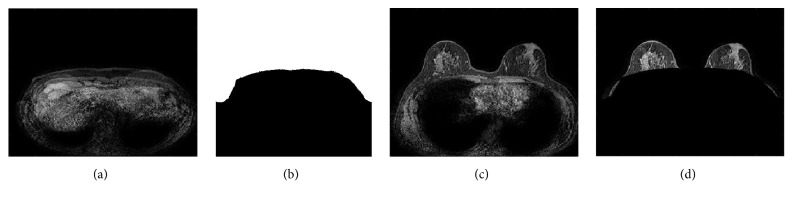
Preprocessing: (a) first slice image of the sequence; (b) mask; (c) generic chest slice (original); (d) breast section slice.

Before computing any measurement on DCE-MRI scans, a preliminary preprocessing is carried out in order to extract the breast section from the chest into all the slices of each temporal scan. This step is performed by considering the first slice of the sequence, which likely represents the chest section, as a mask to subtract from all the other slices in the sequence (Figure 2 reports a sample of the process).

**Figure 3:**
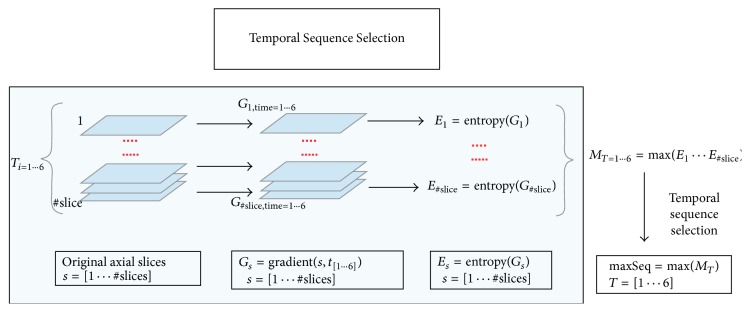
General scheme of the proposed approach: temporal acquisition selection.

**Figure 4:**
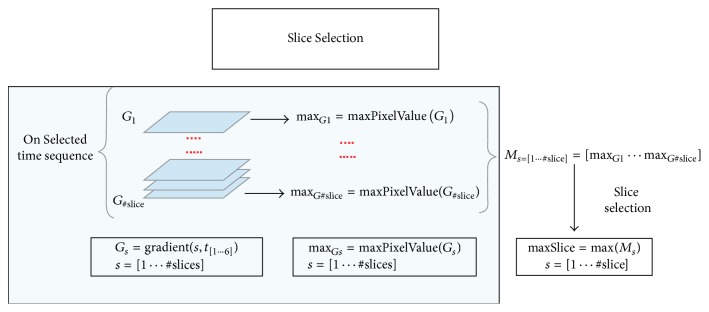
General scheme of the proposed approach: slice selection.

Successively, the gradient of each slice along the temporal dimension is computed. This process will allow making the changes in contrast along the temporal scans more evident. Furthermore, in order to have a synthetic measurement able to synthesize the informative power of each slice, the entropy is computed on each of the 150 axial slices of the sequence. Such a process is iterated on each of the six dynamic acquisitions with the aim of selecting the temporal acquisition in which the gain in the information, that is, the variability over the time, represented by the entropy values is maximized (Figure 3). The temporal acquisition selection will allow in turn selecting the more informative slice among the 150 which is likely to contain a region whose variability over the time is higher with respect to the other slices. Thus, once temporal sequence was fixed, the most informative slice was obtained by computing the maximum gradient on the whole sequence (Figure 4).

**Figure 5:**
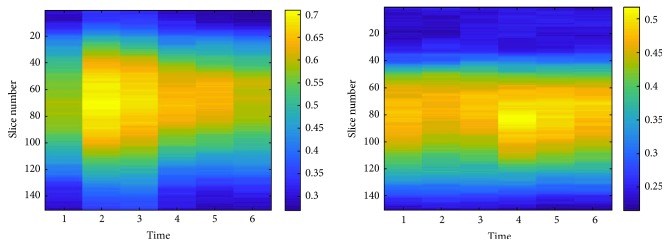
Graphical view of the entropy values computed from the directional gradient images. The color bar indicates the entropy value of each slice in the six temporal acquisitions.

**Figure 6:**
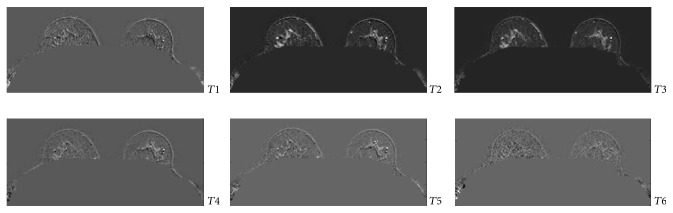
Sample gradient images: the gradient of a slice *x* along the time dimension (*G*_*x*,time=1⋯6_) in the six dynamic acquisitions *T*_1_ ⋯ *T*_6_.

Figures 5 and 6 show the entropy values for each slice for each of the six acquisitions and the directional gradient of a slice along the acquisitions, respectively. It can be noted how much informative, according to the entropy value, are some slices with respect to the others in the same acquisition and with respect to the same slice number in different acquisitions.

Although the gradient and the entropy allow globally evaluating the scans in the temporal acquisition, they alone are not able to provide local information on the single slice that could represent a distinction among different breast regions. For this reason, the mean and standard deviation were introduced as further criteria to achieve discrimination among lesions and different tissue typologies inside the slice that was identified as the one containing “that certain something” of difference. In detail, for each patient, two synthetic images, mean and standard deviation, are created, whose analysis enables providing a remark on different regions to distinguish patients with or without a pathology. Indeed, if both the synthetic images show peaks, the patient is considered to be suffering from a pathology; otherwise, she was considered healthy.

## 3. Results and Discussion

The proposed approach represents a preliminary investigation towards the full automatized analysis of DCE-MRI for breast cancer identification with the aim of increasing diagnosis accuracy even when the parenchymal background enhancement phenomenon makes radiologists examination difficult. In this first study, the potential of the method was proven by the encouraging results obtained in classifying the image with the breast lesion with an accuracy of 82.6%, a sensitivity of 87.0%, and a specificity of 78.3%. As summarized in Table 4, the method seems to be able to distinguish lesions even in patients with moderate parenchymal background.

A first consideration is about the identification of the temporal acquisition characterized by a greater informative power. Indeed, as described in the previous section, the proposed method is able to identify the temporal acquisition in DCE-MRI which maximizes the entropy of the directional gradient image. Specifically, the automatic identified temporal sequence is the same indicated by radiologists: the most informative dynamic acquisitions are obviously the second and the third, that is, the first and second postcontrast times. This identification in turn enables the generation of two further synthetic images able to supply additional information useful in distinguishing between patients with and without lesions.

**Figure 7:**
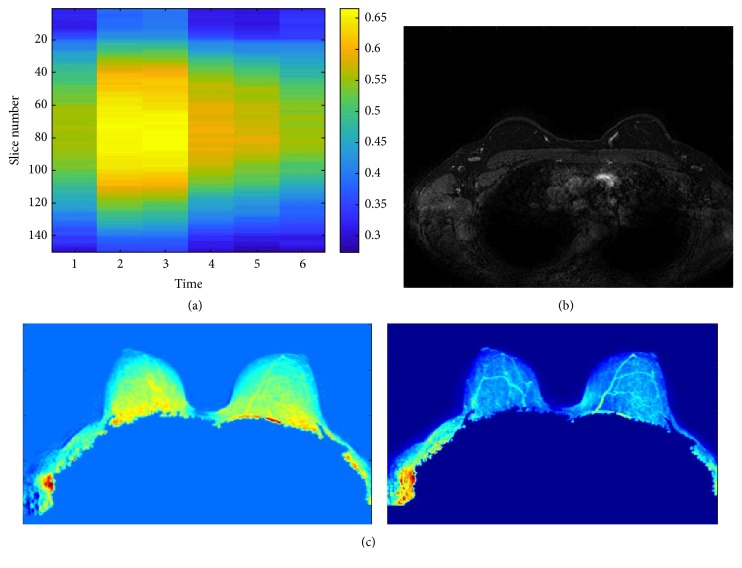
Healthy patient with a moderate parenchymal background (~60%). (a) Representation of the entropy values of directional gradient images of each slice in each scan of the sequence. (b) Slice with the highest gradient in the temporal acquisition *t* = 3, that is, second postcontrast time. (c) Synthetic images of mean and standard deviation.

Figure 7 shows a representation of the entropy values for the directional gradient images of each slice in each scan of the sequence (Figure 7(a)) and the synthetic images of the mean and standard deviation (Figure 7(c)). The MRI examination shows a micronodular pattern with a uniform background without any suspect signs. The temporal acquisition *t* = 3 (i.e., second postcontrast time) was the most informative one (Figure 7(b)). It is possible to observe that the synthetic images of the mean and standard deviation for time *t* = 3 do not show suspicious regions.

**Figure 8:**
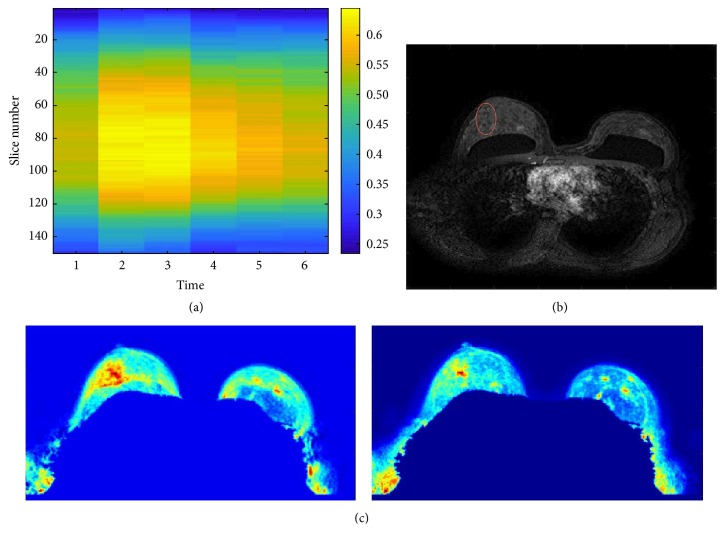
Ill patient with a moderate parenchymal background (~60%) with a non-mass-like lesion in the upper outer quadrant of the right breast. (a) Representation of the entropy values of directional gradient images of each slice in each scan of the sequence. (b) Slice with the highest gradient in the temporal acquisition, *t* = 3, that is, second postcontrast time. The red circle locates the automatically identified lesion. (c) Synthetic images of mean and standard deviation.

The case study reported in Figure 8 refers to a patient whose examination has highlighted a distortion in the upper outer quadrant of the right breast. According to the radiologist, this examination was affected by a particularly high parenchymal background, about 60%. Also in this case, the most informative temporal acquisition obtained by the analysis of the entropy values of the directional gradients (Figure 8(a)) resulted to be *t* = 3, that is, second postcontrast time. Interestingly, the synthetic images of the mean and standard deviation made it possible to locate the lesion. Indeed, it is possible to note that the synthetic images of the mean and standard deviation for temporal sequence *t* = 3 highlighted a region of interest in correspondence of the lesion (Figure 8(c)). This is an important remark confirming that the proposed approach could be effectively exploited as radiologists support. A further consideration about the potentiality of the approach is about the individuation of the slice, among the whole sequence in the selected temporal acquisition, which corresponds to the indication of the radiologist that signed the slice as the one in which the lesion is more visible.  Figure 8(b) shows the slice at time *t* = 3 containing the automatically identified lesion, in accordance with the radiologists' indications. It is possible to note that the lesion is not easily detectable due to the high parenchymal background.

**Figure 9:**
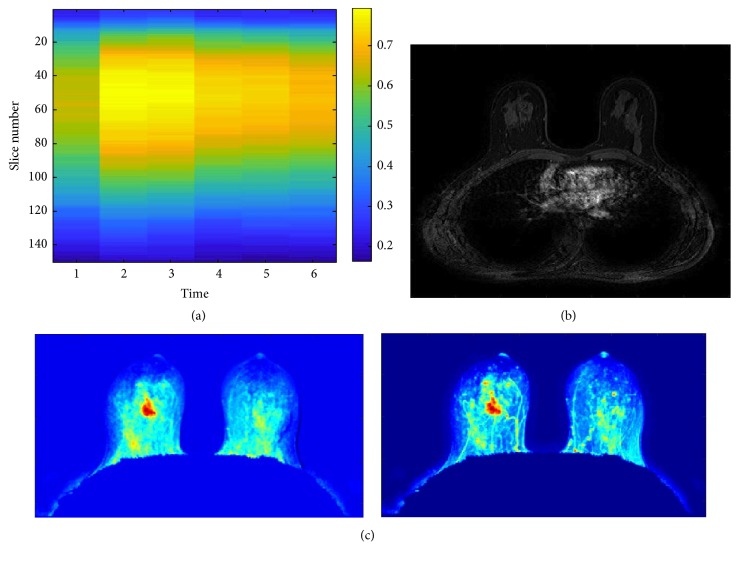
Patient with a moderate parenchymal background (~70%) with MR examination doubtful. (a) Representation of the entropy values of directional gradient images of each slice in each scan of the sequence. (b) Slice with the highest gradient in the temporal acquisition, *t* = 2, that is, first postcontrast time. (c) Synthetic images of mean and standard deviation.

However, within the analyzed sample data, there were some misclassifications that deserve appropriate further study. As an example, the images of the mean and standard deviation in Figure 9(c), which synthesize the selected temporal sequence *t* = 2 (Figure 9(b)), show a suspicious region in a patient with a moderate parenchymal background and whose examination was doubtful. Really, the MR examination highlighted multiple areas with non-mass-like contrasting joints probably due to the hormonal stimulation. Note that these doubtful cases were not counted in the calculation of accuracy.

## 4. Conclusion

Breast cancer is currently the most common cancer in women. Early detection by imaging studies becomes the key point for improving breast cancer prognosis. Breast MRI is usually used for screening in high-risk women and for determining the extent of disease. In literature, the attention of the scientific community is addressed to solve diagnostic issues of breast diseases. Nevertheless, also the localization of the lesion can be difficult for the radiologists. In this context, our innovative model aims to detect the lesions. At MRI, both normal and abnormal breast tissues enhance after contrast material administration, a phenomenon known as background parenchymal enhancement. In this study, we proposed and evaluated the efficacy of some statistical measurements to automatically characterize the enhancement trend of the whole breast area. We evaluated the accuracy of the proposed approach to discriminate normal and abnormal tissues, even when the BPE degree is high.

In developing our model, the radiologists only intervened in the initial phase to locate any lesions; this information was used to test the method that worked automatically and blindly. Preliminary experimental evaluations show the potentiality of the proposal in detecting breast lesions, especially in patients with a mild or moderate degree of background parenchymal enhancement, and the accuracy exceeds 75%. It should be emphasized that, in this preliminary phase of the work, we have not automated the localization of the lesion in synthetic images, because the cases analyzed are numerically reduced. However, the peak value provides a first indication of the location of the lesion in each of the two synthetic images, and, in the future works, this information can be used to automate the procedure to localize the lesion.

## Figures and Tables

**Table 1:** MRI acquisition parameters.

	STIR-TSE	T2-weighted TSE	T1-weighted 3D-DCE
TR (ms)	3800	6.300	4.4
TE (ms)	60	130	2.0
TI (ms)	165	––	––
FOV ((AP × RL mm))	250 × 450	250 × 450	250 × 450 × 150 (FH)
Matrix size	168 × 300	336 × 600	168 × 300
Partitions	50	50	100
Slice thickness (mm)	3	3	1.5
Intersection gap	0	0	––
Signal avg.	3	3	––
Turbo factor	23	59	50
SENSE factor	––	1.7	1.6
Voxel size (mm^3^)	1.5 × 1.5 × 3.0	0.75 × 0.75 × 3.0	––

**Table 2:** Items of evaluation and classification.

Points	0	1	2
Shape	Round	Dendritic	—
	Oval	Irregular	
Border	Well-defined	Ill-defined	—
CM patterns	Homogeneous	Heterogeneous	Rim
Initial enhancement	<50%	50–100%	>100%
Postinitial enhancement	Continuous increase	Plateau	Washout

**Table 3:** Classification of the score.

Group	Points	Diagnostic value
I	0-1	Benign
II	2	Probably benign
III	3	Probably benign
IV	4-5	Suspicious abnormality
V	6–8	Highly suggestive of malignancy

**Table 4:** Performances of each BPE category.

BPE	Number of cases	Age distribution (%)			Performances (%)		
Categories	(normal/abnormal)	≤45	45–55	>55	Acc	Se	Sp
Minimal	20 (11/9)	45.0	35.0	20.0	95.0	100.0	90.9
Mild	18 (8/10)	27.8	50.0	22.2	77.8	80.0	75.0
Moderate	8 (4/4)	62.5	37.5	–	75.0	75.0	75.0

Total	46 (23/23)	43.5	39.1	17.4	82.6	87.0	78.3
